# MEN1 redefined, a clinical comparison of mutation-positive and mutation-negative patients

**DOI:** 10.1186/s12916-016-0708-1

**Published:** 2016-11-15

**Authors:** Joanne M. de Laat, Rob B. van der Luijt, Carolina R. C. Pieterman, Maria P. Oostveen, Ad R. Hermus, Olaf M. Dekkers, Wouter W. de Herder, Anouk N. van der Horst-Schrivers, Madeleine L. Drent, Peter H. Bisschop, Bas Havekes, Menno R. Vriens, Gerlof D. Valk

**Affiliations:** 1 Department of Endocrine Oncology, University Medical Center Utrecht, Utrecht, The Netherlands; 2 Department of Medical Genetics, University Medical Center Utrecht, Utrecht, The Netherlands; 3 Department of Endocrinology, Radboud University Medical Center, Nijmegen, The Netherlands; 4 Departments of Endocrinology and Metabolism & Clinical Epidemiology, Leiden University Medical Center, Leiden, The Netherlands; 5 Department of Internal Medicine, Erasmus Medical Center, Rotterdam, The Netherlands; 6 Department of Endocrinology, University Medical Center Groningen, Groningen, The Netherlands; 7 Department of Internal Medicine, VU University Medical Center, Amsterdam, The Netherlands; 8 Department of Endocrinology and Metabolism, Academic Medical Center, Amsterdam, The Netherlands; 9 Department of Internal Medicine, Division of Endocrinology, Maastricht University Medical Center, Maastricht, The Netherlands; 10 Department of Surgery, University Medical Center Utrecht, Utrecht, The Netherlands

**Keywords:** MEN1, Diagnosis, Survival

## Abstract

**Background:**

Multiple Endocrine Neoplasia type 1 (MEN1) is diagnosed when two out of the three primary MEN1-associated endocrine tumors occur in a patient. Up to 10–30 % of those patients have no mutation in the *MEN1* gene. It is unclear if the phenotype and course of the disease of mutation-negative patients is comparable with mutation-positive patients and if these patients have true MEN1. The present study aims to describe and compare the clinical course of *MEN1* mutation-negative patients with two out of the three main MEN1 manifestations and mutation-positive patients during long-term follow-up.

**Methods:**

This is a cohort study performed using the Dutch MEN1 database, including > 90 % of the Dutch MEN1 population.

**Results:**

A total of 293 (90.7 %) mutation-positive and 30 (9.3 %) mutation-negative MEN1 patients were included. Median age of developing the first main MEN1 manifestation was higher in mutation-negative patients (46 vs. 33 years) (*P* = 0.007). Mutation-negative patients did not develop a third main MEN1 manifestation in the course of follow-up compared to 48.3 % of mutation-positive patients (*P* < 0.001). Median survival in mutation-positive patients was estimated at 73.0 years (95 % CI, 69.5–76.5) compared to 87.0 years (95 % CI not available) in mutation-negative patients (*P* = 0.001).

**Conclusions:**

Mutation-positive and mutation-negative MEN1 patients have a different phenotype and clinical course. Mutation-negative patients develop MEN1 manifestations at higher age and have a life expectancy comparable with the general population. The apparent differences in clinical course suggest that *MEN1* mutation-negative patients do not have true MEN1, but another MEN1-like syndrome or sporadic co-incidence of two neuro-endocrine tumors.

**Electronic supplementary material:**

The online version of this article (doi:10.1186/s12916-016-0708-1) contains supplementary material, which is available to authorized users.

## Background

Multiple Endocrine Neoplasia type 1 (MEN1) is a rare autosomal inherited disorder with an estimated prevalence of 1–10/100,000 and is characterized by the occurrence of three primary manifestations: primary hyperparathyroidism (pHPT), duodenopancreatic neuroendocrine tumors (dpNET) and pituitary tumors (PIT) [1, 2]. According to the present clinical practice guidelines [3], MEN1 is diagnosed based on clinical, familial or genetic criteria. On the basis of clinical criteria, MEN1 is diagnosed if at least two out of the three primary MEN1 manifestations occur in a patient. MEN1 is diagnosed on the basis of familial criteria if a patient has a MEN1 manifestation in combination with a first degree family member with MEN1. The identification of a germline MEN1 mutation in an individual who may be asymptomatic also confirms the diagnosis of MEN1.

In 10–30 % of patients who were diagnosed with MEN1 based upon clinical criteria, no mutation was found in the *MEN1* gene [4–6]. These so called ‘phenocopies’ are an upcoming diagnostic challenge. With the increased use and improvement of diagnostic techniques, the incidence and prevalence of pHPT and micro adenoma of the pituitary gland is rapidly rising. In a population-based study [7], the prevalence of pHPT tripled during the past two decades, increasing from 76 to 233 per 100,000 women and from 30 to 85 per 100,000 men. With an magnetic resonance imaging (MRI) scan, which is now widely available, small pituitary adenomas were found in as many as 10 % of healthy volunteers [8]. These numbers implicate that a large number of patients might strictly meet the clinical criteria for MEN1 because of the co-incidence of pHPT and pituitary adenoma. However, it is questionable whether those patients are at risk of developing other MEN1-associated tumors and will benefit from intensive lifelong screening for MEN1-related manifestations.

Recently, research concerning other genes has been performed trying to explain the MEN1 clinical phenotype in cases of a mutation-negative MEN1 syndrome [9, 10]. In a small series of patients, a small proportion of the mutation-negative MEN1 patients appeared to have a mutation in the *CDKN1B* gene [11]. These patients typically present with pituitary and parathyroid tumors. The course of the disease appeared also to be different in these patients for whom the term MEN4 was introduced [12].

For MEN1 patients, once the syndrome is diagnosed, the early detection of MEN1-associated tumors and subsequent interventions seem to lead to a more favorable course of the disease with a subsequent improved survival [13, 14]. Consequently, the current guidelines advise an intensive follow-up of patients with frequent laboratory and radiological investigations for all patients irrespective of age [3]. It is, however, unclear if mutation-negative patients are at risk for developing further MEN1-related (neuro-)endocrine tumors and benefit equally from this intensive follow-up as mutation-positive patients do. To date, data on the penetrance of clinical manifestations and survival of MEN1 patients, irrespective their mutational status, are based on single- or sometimes multi-institution studies of mainly tertiary referral centers [15–20], which may have led to a selection of patients included in the studies.

Therefore, the aim of the present study was to compare the long-term clinical course of the disease in *MEN1* gene mutation-positive patients with *MEN1* gene mutation-negative patients from the national Dutch MEN1 cohort, including > 90 % of the MEN1 population. We studied incidence, age-related penetrance of MEN1-related manifestations, and survival in both groups of patients. Furthermore, the mutation-negative patients were invited to undergo additional genetic testing, including assessment of mutations in the *CDKN1B* gene.

## Methods

### Study design and patients

The study was performed using data from the Dutch MEN1 study group database. This longitudinal database includes > 90 % of all Dutch MEN1 patients, aged 16 years and older at the end of 2010, treated at one of the Dutch University Medical Centers (UMCs) between 1990 and 2011 [21]. Data of all identified patients were collected according to a predefined protocol, which was based on predefined study questions from every quarter of every available year of follow-up during the period 1990–2014. The study protocol was approved by the Medical Ethical Committees of all UMCs in the Netherlands. Given the retrospective and observational data of the study, the use of these clinical data, including the results of *MEN1* gene testing, were approved for the study aims and the requirement to obtain informed consent was waived. For the additional genetic analyses of *CDKN1B* and *AIP* genes in mutation-negative patients, oral and written informed consent was obtained for the testing and the use of the results in this study.

We assessed follow-up of patients with a known *MEN1* gene mutation (mutation-positive) and patients with a negative *MEN1* gene mutation test who had two out of the three primary MEN1 manifestations (mutation-negative).

### Definitions of MEN1 manifestations

We defined pHPT as hypercalcemia combined with elevated or inappropriately non-suppressed parathyroid hormone levels in two consecutive measurements.

The reference test for the presence of pancreatic neuroendocrine tumor (NET) was the outcome of pathology examination. If pathology was not available, pancreatic NET presence was based on MRI, computed tomography (CT) or endoscopic ultrasound, which had to be confirmed at least once by consecutive imaging studies. The absence of pancreatic NET also had to be confirmed on a minimum of two subsequent imaging studies during follow-up [22]. A duodenal NET was diagnosed with gastroduodenoscopy and, if available, by pathology. A dpNET was diagnosed according to the reference standard of pancreatic NET and duodenal NET. Thymic-NET was diagnosed based upon the results of pathology examination. Patients were considered to have a lung NET if (1) pathology examination showed lung NET or (2) radiological examination was positive for lung NET, as previously described [23]. Gastric NET was diagnosed by gastroduodenoscopy and had to be confirmed by pathology.

The reference standard for the presence of PIT was (1) pathology or (2) radiological examination demonstrating a PIT. Details for the reference standard of pit have been described previously [24].

An adrenal tumor (ADR) was diagnosed based on pathology, and if pathologic examination was not available, on radiological imaging (CT or MRI) which had to be confirmed on subsequent imaging.

### Genetic analysis

DNA was extracted from peripheral blood. The presence of a *MEN1* mutation was determined with DNA sequencing since 1998 and with a combination of DNA sequencing and multiplex ligation-dependent probe amplification (MLPA) since 2005. MLPA (MRC-Holland, Amsterdam, The Netherlands) is used for detecting large deletions or duplications in the *MEN1* gene. In addition, mutation-negative patients were invited to participate in a genetic screening program for familial NETs (including *CDKN1B* and *AIP*). The presence of a *CDKN1B* and *AIP* mutation was determined by direct sequencing. In addition, MLPA was used for *AIP* to detect large deletions or duplications. Sequences were analyzed with Sequencing Analysis software version 5.2 (Applied Biosystems) and compared with the reference sequences of each gene (Ensembl identifiers: *MEN*1 gene EST00000312049; *CDKN1B* gene: NM_004064.4 and *AIP* gene: ENST00000279146) using SeqScape software version 2.5 (Applied Biosystems). Primer sequences and PCR conditions are available on request.

### Outcome measures

The primary outcomes of the study were the age-related penetrance of the MEN1 manifestations. The secondary outcomes were the incidence of MEN1 manifestations per 1000 patient-years and the survival of patients. The primary and secondary outcomes were compared between the mutation-positive and mutation-negative patient groups.

### Statistical analysis

Age-related penetrance of the primary MEN1 manifestation and other MEN1-associated tumors was estimated using the Kaplan–Meier method. Age-related penetrance was calculated for the age per manifestation, and for the age on which a first, second, and third major MEN1 manifestation was diagnosed.

Subgroup analyses for mutation-positive and mutation-negative patients was performed for the age of first, second, and third major MEN1 manifestation and the occurrence of the first other MEN1-associated tumor. In addition, a subgroup analyses was performed comparing patients in whom MEN1 was diagnosed because of the occurrence of two of the primary MEN1 manifestations (index patients) who were mutation-positive on the one hand and mutation-negative patients on the other. Index patients were not siblings of a known MEN1 family before clinical diagnosis of MEN1 and testing. Comparison of Kaplan–Meier curves was made using the Log-rank test.

Survival was compared for mutation-positive and mutation-negative patients using the Kaplan–Meier method. Cause of death was subdivided in MEN1 related and non-MEN1 related. Comparison was made using the Log-rank test. Cause and age of death was compared between mutation-positive and mutation-negative patients.

Incidence rate was calculated for the three primary MEN1 manifestations. For calculation of incidence, only tumors detected at follow-up of patients with MEN1 syndrome, i.e. after establishing the MEN1 diagnosis, were considered.

To describe clinical characteristics, the mean ± SD or median with range was calculated, depending on the normal distribution. Continuous variables were analyzed by using independent sample t-test or Mann–Whitney U test. Dichotomous variables were compared with Fisher exact test or χ^2^ test.

Statistical significance was set at *P* < 0.05 and the analyses were conducted using SPSS 20.0.

## Results

### Study population

In the period 1990–2011, a total of 322 MEN1 patients were included in the database. There was a female predominance (n = 187, 58.1 %) and the patients were part of 121 different MEN1 families. At the moment of genetic testing, 100 patients were diagnosed based on clinical grounds and those patients were not siblings of a known MEN1 family. These patients represent the subgroup of index patients, 70 (70 %) of whom tested positive and 30 (30 %) tested negative for the *MEN1* mutation. The other patients were all mutation-positive siblings of already known MEN1 families; 91 were pre-symptomatic at testing, and 131 patients were tested after development of the first MEN1 manifestation. The total number of *MEN1*-positive patients was 292 (90.7 %).

The median age of diagnosis of MEN1 was 37 years (range 8–78). In *MEN1* gene mutation-negative patients, median age of diagnosis of MEN1 was 55 years (range 23–78), and was 20 years higher than the age of diagnosis of mutation-positive MEN1 patients (median age MEN1 diagnosis 35 years (range 8–78 years); *P* < 0.001).

### Clinical manifestations

**Fig. 1:**
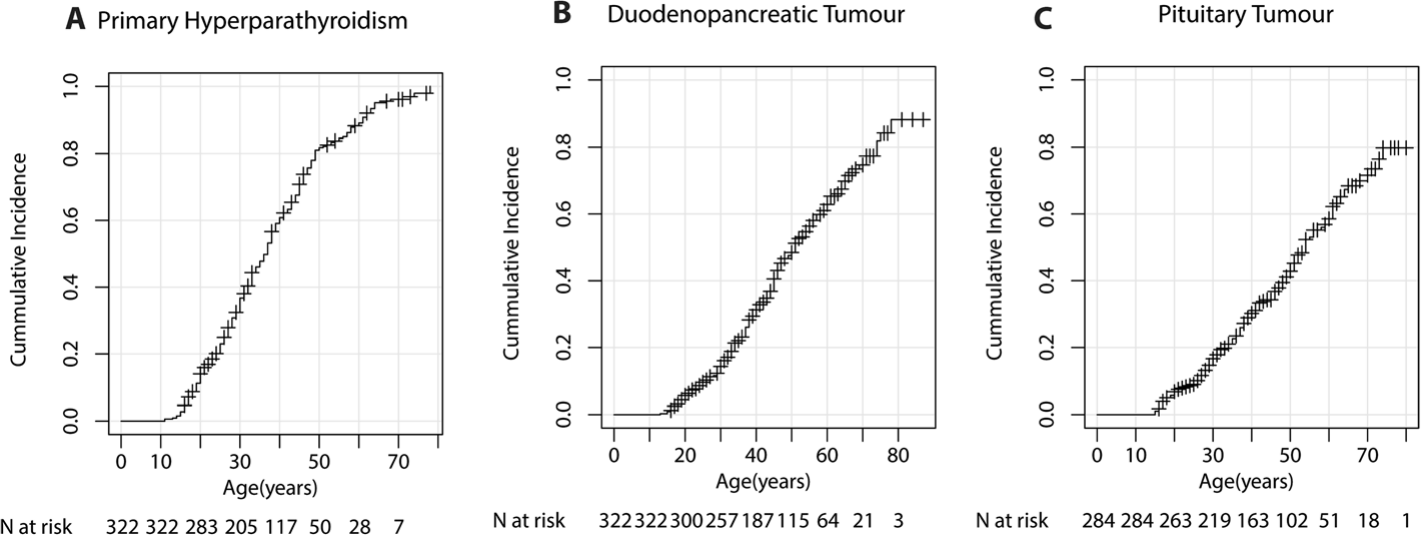
Age-related penetrance of the main MEN1 manifestations. **a** Age-related penetrance of duodenopancreatic neuroendocrine tumors. **b** Age-related penetrance of pituitary tumors. **c** Age-related penetrance of primary hyperparathyroidism

In 92 % of the patients, at least one of the three main MEN1 manifestations was diagnosed. pHPT occurred in 87.0 % of patients and was diagnosed at a mean age of 36 years (SD 14 years). The youngest patient diagnosed with pHPT was 11 years old. dpNET occurred in 55.9 % and PIT in 43.8 %. The mean age at diagnosis of dpNET and PIT was 41 years (SD 15 years) and 40 years (SD 15 years), respectively. Age-related penetrance for the three main manifestations is presented in Fig. 1a–c. The prevalence of other manifestations associated with MEN1 was gastric NET in 3.7 %, lung NET in 19.3 %, thymic NET in 4.0 %, and ADR in 31.4 % of patients (Table 1). Median age and range for all manifestations are presented in Additional file 1: Table S1.

**Table 1:** Prevalence of MEN1 manifestations

MEN1 manifestations	Total	Mutation-positive	Mutation-negative
	n (%)	n (%)	n (%)
Number of patients	322	292	30
Primary hyperparathyroidism	280 (87.0)	252 (86.0)	28 (93.3)
Duodenopancreatic neuroendocrine tumors	180 (55.9)	173 (59.2)	7 (23.3)
Pituitary tumor	141 (49.6)	116 (39.7)	25 (83.3)
Lung neuroendocrine tumor	62 (19.3)	62 (21.2)	0
Thymic neuroendocrine tumor	13 (4.0)	13 (4.5)	0
Gastric neuroendocrine tumor	12 (3.7)	12 (4.1)	0
Adrenal tumor	101 (31.4)	100 (34.1)	1 (3.3)

### Incidence of primary MEN1 manifestations

Primary hyperparathyroidism was predominantly diagnosed before MEN1 was diagnosed. Therefore, the incidence of pHPT during follow-up was ‘only’ 144.5 per 1000 patient-years (95 % confidence interval (CI), 113.3–181.8; 73 new cases in 119 patients followed 505 patient-years). Incidence of PIT was 39.6 per 1000 patient-years (95 % CI, 31.7–48.9; 87 new cases in 230 patients followed 2195 patient-years). The incidence of dpNET was 62.3 per 1000 patient-years (95 % CI, 51.8–74.3; 124 new cases in 241 patients followed for 1990 patient-years.

### Age-related penetrance

**Fig. 2:**
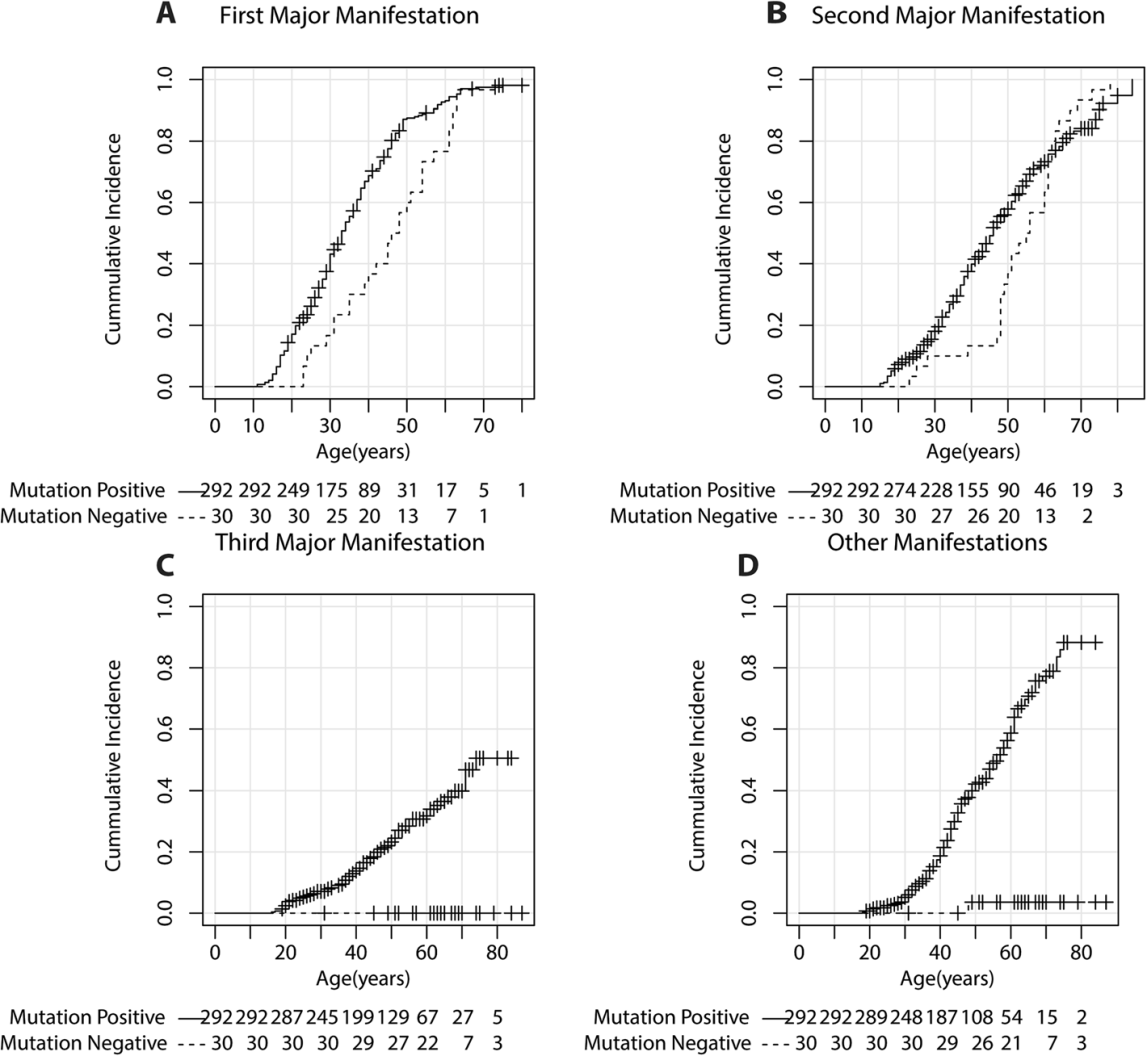
Age-related penetrance of major manifestations and other MEN1-associated tumors compared between mutation-positive patients and mutation-negative patients. **a** Age-related penetrance of the first manifestation (Log-rank test *P* = 0.007). **b** Age-related penetrance of the second manifestation (Log-rank test *P* = 0.559). **c** Age-related penetrance of the third manifestation (Log-rank test *P* < 0.001). **d** Age-related penetrance of other MEN1-associated neuroendocrine tumors (Log-rank test *P* = 0.003)

The median age at which patients developed the first major manifestation was 13 years higher in *MEN1* mutation-negative patients: 46.0 years (95 % CI, 39.6–52.4) compared to 33.0 years (95 % CI, 30.9–35.1) in *MEN1* mutation-positive patients (*P* = 0.007) (Fig. 2a). Median age at developing the second major manifestation was 9 years higher in mutation-negative patients; however, the difference was not significant, 55.0 years (95 % CI, 48.3–61.7) in *MEN1* mutation-negative patients versus 46.0 years (95 % CI, 43.3–48.7) in mutation-positive patients (*P* = 0.559) (Fig. 2b). A third main manifestation did not develop in *MEN1* mutation-negative patients, compared to a total of 76 in the mutation-positive patient group, corresponding with a median age of 72 for developing the third manifestation (*P* < 0.001) (Fig. 2c). Finally, other MEN1-associated manifestations developed in only one patient who was *MEN1* mutation-negative (ADR), while 48.3 % of mutation-positive patients developed such manifestations at a median age of 57.0 (53.5–60.5) (Fig. 2d).

Comparable results were found when the subgroup of index patients and mutation-negative patients were compared (Additional file 1: Figure S1). In this subgroup analysis, the mean age at diagnosis of the second major manifestation in mutation-positive index cases was 38.0 years (95 % CI, 35.8–40.2), which is 17 years younger compared to mutation-negative patients (*P* = 0.003). A third main manifestation was found in 32 mutation-positive index cases at a median age of 65 years.

### Survival

**Fig. 3:**
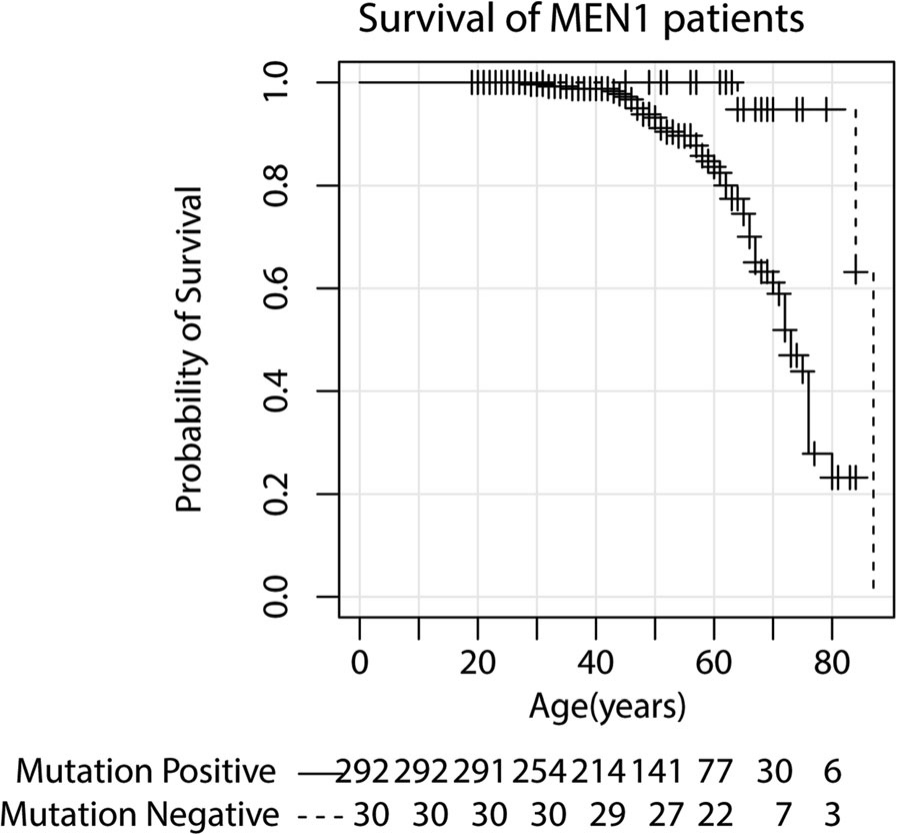
Survival curve of MEN1 patients, comparing between mutation-positive and mutation-negative patients

After a median follow-up period from the moment of MEN1 diagnosis of 10 years (range 0–47 years), 54 patients died (16.8 %). Cause of death can be found in Additional file 1: Table S2. Among the mutation-positive MEN1 patients, 51 died (17.5 %), of whom 30 (58.8 %) due to a MEN1-related cause. The mean age of death was 60 years (SD 12 years). Among mutation-negative patients, three patients died (10.0 %) from causes not related to MEN1. Survival curves are presented in Additional file 1 (Fig. 3). Median survival in mutation-positive patients was estimated at 73.0 years (95 % CI, 69.5–76.5) compared to 87.0 years (95 % CI not available) in mutation-negative patients (*P* = 0.001).

Survival in mutation-positive index patients was comparable with the survival in other mutation-positive cases. Median survival in mutation-positive index cases was estimated at 72.0 years (95 % CI, 67.0–77.0; Additional file 1: Figure S2). A total of 22 mutation-positive index patients died during follow-up, 16 (72.7 %) of whom due to a MEN1-related cause (Additional file 1: Table S4).

### Additional genetic results in mutation-negative patients

Full results of manifestation and additional genetic testing in *MEN1* mutation-negative patients are summarized in Additional file 1: Table S3. In the 30 mutation-negative patients, no family members with MEN1 were identified. A pHPT/PIT phenotype was present in 23 patients (76.7 %), five patients had pHPT/dpNET (16.7 %) and two had PIT/dpNET (6.7 %).

In 21 of 27 patients (77.8 %) who were still alive, permission was obtained for additional genetic analysis, one patient could not give permission because of the presence of dementia. In all 21 patients, genetic testing for *CDKN1B* had been performed, which revealed a *CDKN1B* mutation in one patient. This patient, who was diagnosed with pHPT and PIT, had a mutation in the *CDKN1B* gene (c.295_305dup p.Gln104Argfs*19). At this location a duplication of 11 base pairs was detected in exon 1, causing a frame shift starting at codon Gln104. The new reading frame ends in a STOP codon 18 positions downstream. In 19 out of 21 patients, genetic testing for AIP was performed, which did not result in additional mutations.

## Discussion

In the Netherlands, MEN1 patients have an increased risk of premature death with an estimated median survival of 73 years, which is almost 10 years shorter compared with the general Dutch population. *MEN1* mutation-negative patients appeared to have a far less aggressive course of disease. In mutation-negative patients, the age of the first MEN1 manifestation was higher, and a third MEN1-related manifestation hardly ever occurred. A striking result is the difference in age of death and median survival when *MEN1* mutation-positive patients are compared with mutation-negative patients. The shorter life expectancy of mutation-positive MEN1 patients is in line with previous literature. The apparent differences in penetrance of MEN1 manifestations and survival between *MEN1* mutation-negative and positive patients indicate that *MEN1* mutation-negative patients with two out of the three main MEN1 manifestations have a different MEN1 mimicking disease. Additional testing for *CDKN1B* mutations in our national MEN1 cohort led to one positive, not previously described, mutation underlining the rarity of *CDKN1B* mutations as explanation for the MEN1 phenotype.

### Strengths

To minimize selection bias, age-related penetrance was analyzed in the national Dutch MEN1 study group cohort of MEN1 patients, which includes more than 90 % of the total Dutch MEN1 population [21]. The follow-up data was collected based on pre-defined study questions and according to a standardized protocol for every quarter of every year from 1990 up to 2011. The database is considered of high quality for its high density of reliable data and long-term follow-up. To our knowledge for the first time, the age-related penetrance and age of death were compared between mutation-positive and mutation-negative patients. Diagnosis of a MEN1 manifestation was established through predefined reference standards to increase reliability. MEN1 manifestations diagnosed only on imaging had to be confirmed on consecutive examinations leading to a valid diagnosis. Further, assessment of the incidence of newly diagnosed manifestations over time is relatively new in the MEN1 research field. In this study, additional testing for mutations in the *CDKN1B* gene was performed and the finding of only one positive test underlines the rarity of these mutations when also tested in a national cohort.

### Comparison with the other literature

The prevalence of the major MEN1-related manifestations, age-related penetrance and the survival rates are comparable with the present literature [19, 20, 25]. The previously reported percentages of mutation-negative patients vary, but our 9.3 % compares low to some earlier studies [3, 5, 6]. Moreover, in previous studies, more than 50 % of index patients without a family history of MEN1 were mutation-negative, compared to 30 % in our study [6, 26–28]. The mutation-negative patients included in our database generally underwent the recently implemented MLPA analysis, which increased the sensitivity of *MEN1* gene analysis leading to a lower proportion of *MEN1* mutation-negative patients. In another recent study, a comparable rate of 10 % mutation-negative patients was identified [4].

Previous studies have reported that mutation-negative patients are predominantly index patients without a family history of MEN1 [6, 26–28]. In our cohort all mutation-negative patients were index patients, confirming the results of previous reports. In some reports it was already suggested that patients scoring negative on both mutational testing and familial history more often have mild clinical presentation [26, 28]. We have now demonstrated that the differences in the clinical course of mutation-positive and negative patients are lasting at long-term follow-up. Index patients who scored positive at mutational testing, however, have a comparable course of the disease as patients with familial occurrence of MEN1. The differences in clinical course suggest that the mutation-negative patients may not have the MEN1 syndrome.

Recently, a new germ-line mutation in the *CDKN1B* gene [11] was discovered in mutation-negative MEN1 patients, which is now identified as MEN4. Incidence and clinical implications of MEN4 syndrome are still unknown [12]. Mutations of the *MEN1* gene and *CDKN1B* polymorphisms can also coincide, resulting in early development of aggressive tumors in MEN1 patients [29]. In our cohort, permission for additional genetic analysis was obtained from 21 of 27 live patients (77.8 %). Only one *CDKN1B* mutation was found in all tested patients. Our findings are in accordance with the few studies published in which sporadic and familial cases of mutation-negative MEN1 patients are tested for *CDKN1B* [5, 30–33]. These studies also show that mutations in *CDKN1B* and the other genes coding CDKIs are extremely rare, and many patients who are mutation-negative and have a MEN1 like disease still have a genetically unexplained phenotype.

The *CDKN1B* mutation, which we found in one patient, had not been described before. The duplication of 11 base pairs causes a frame shift, resulting in a STOP codon. The mRNA produced might be targeted for non-sense mediated decay. We assume that this is a pathogenic mutation, because of the presence of an STOP codon. A number of somatic mutations in exon 1 of the *CDKN1B* gene are described.

Strictly spoken, we might have missed mutation-negative patients with two main MEN1-associated tumors who were not referred to one of the UMCs. However, in the Dutch healthcare system, especially patients with a lower age of diagnosis of manifestations or a more aggressive course of the disease are generally referred to one of the UMCs. Missing patients who were not referred can therefore be expected to have led to an underestimation of the differences between the *MEN1* mutation-positive and -negative patients underlining the validity of the results. Finally, in The Netherlands, all genetic tests for MEN1 are performed only at the genetic laboratory of the UMC Utrecht, leading to completeness of the data. Extrapolating the clinical data from the application forms for the genetic tests that led to negative results led to the conclusion that the majority of mutation-negative patients had been included [21].

### Limitations

Nine of the 30 mutation-negative patients were not tested for *CDKN1B*. These missing data could lead to an underestimation of the incidence of *CDKN1B* mutations among the *MEN1* mutation-negative patients. However, the low prevalence of *CDKN1B* mutations is in line with previous literature.

At the moment of diagnosis of the first manifestation, the *MEN1* mutation-negative patients were not suspected to have MEN1 by the treating physician. Therefore, routine follow-up and screening as part of the MEN1 protocol started only after diagnosis of the second main manifestation. Thus, age of the second manifestation might be overestimated in the mutation-negative patients. However, the outcomes were confirmed by the results of the additional analysis in which *MEN1* mutation-negative patients were compared with newly diagnosed *MEN1* mutation-positive patients (index patients) who often underwent mutation analyses because of the combination of two MEN1 manifestations and also started structured follow-up after the diagnosis of MEN1.

### Clinical implications

According to our results, the age of MEN1 diagnosis and the age of the MEN1 manifestations are significantly higher in mutation-negative patients with two of the main MEN1-associated tumors. We have now demonstrated that these patients also have a more favorable clinical course, justifying the concept that these patients might not have a true MEN1 syndrome, but a MEN1-like syndrome or sporadic co-incidence of two (neuroendocrine) tumors. According to the favorable clinical course of these patients, it is questionable whether or not these patients should be intensively screened for future endocrine events according to the clinical guidelines for MEN1 patients.

Caution is warranted when encountering a mutation-negative patient who has a positive family history for MEN1 or with three MEN1-related manifestations. In our cohort, all mutation-negative patients were index cases with two main MEN1-related manifestations and a negative family history; however, previous papers have reported negative mutational testing in patients with familial occurring MEN1 [6, 26, 28]. We do expect that cases with a negative mutational testing in combination with a positive family history will become highly uncommon in the present era of highly sensitive tests such as MLPA and next generation sequencing [34, 35].

## Conclusions

In conclusion, MEN1 is a syndrome with high life-time occurrence of pHPT, dpNET, and PIT and a considerable risk of premature death with an estimated median survival of 73 years. *MEN1* mutation-positive and mutation-negative patients have a different clinical course, with mutation-negative patients developing MEN1 manifestations at a higher age and having a median survival comparable to the general population, which is 10 years longer than *MEN1* mutation-positive patients. Mutations in the *CDKN1B* gene can be considered a rarity and do not fully explain the occurrence of MEN1 phenocopies. The apparent differences in clinical course suggests that mutation-negative patients with two out of the three main MEN1 manifestations do not have true MEN1, but another MEN1-like syndrome or sporadic co- incidence of two NETs.

## Additional file

Additional file 1: Table S1. Age of diagnosis of MEN1-associated manifestations. **Table S2.** Causes of mortality. **Table S3.** Clinical characteristics and results of genetic screening. **Figure S1.** Age-related penetrance of major manifestations and other MEN1-associated tumors compared between mutation positive index patients and mutation negative patients. **Figure S2.** Survival curve of MEN1 patients, comparing between mutation-positive and mutation-negative index patients. **Table S4.** Causes of mortality in index cases. (PDF 928 kb)

## Abbreviations

ADR: Adrenal tumor
*AIP*: Aryl hydrocarbon receptor interacting protein
*CDKN1B*: Cyclin-Dependent Kinase Inhibitor 1B
CT: Computed tomography
dpNET: Duodenopancreatic neuroendocrine tumors
MEN1: Multiple Endocrine Neoplasia type 1
MEN4: Multiple Endocrine Neoplasia type 4
MLPA: Multiplex ligation-dependent probe amplification
MRI scan: Magnetic resonance imaging scan
NET: Neuroendocrine tumor
PIT: Pituitary tumors
pHPT: Primary hyperparathyroidism (pHPT)
SD: Standard deviation
UMCs: University Medical Centers
95% CI: 95% confidence interval
