# Supplementary material for: MEN1 redefined, a clinical comparison of mutation-positive and mutation-negative patients
Source: BMC Med. 2016 Nov 15;14:182. doi: 10.1186/s12916-016-0708-1 (PMC5109674; doi:10.1186/s12916-016-0708-1)
Supplement: Additional file 1: Table S1. — Age of diagnosis of MEN1-associated manifestations. Table S2. Causes of mortality. Table S3. Clinical characteristics and results of genetic screening. Figure S1. Age-related penetrance of major manifestations and other MEN1-associated tumors compared between mutation positive index patients and mutation negative patients. Figure S2. Survival curve of MEN1 patients, comparing between mutation-positive and mutation-negative index patients. Table S4. Causes of mortality in index cases. (PDF 928 kb) [file 12916_2016_708_MOESM1_ESM.pdf]

Supplementary appendix

to accompany

" MEN1 redefined: A clinical comparison of mutation- positive and mutation- negative patients with MEN1"

Table of contents

Table S1	Age of diagnosis of MEN1 associated manifestations
Table S2	Causes of mortality
Table S3	Clinical characteristics and results of genetic screening
Figure S1	Age related penetrance of major manifestations and other MEN1-associated tumors compared between mutation positive index patients and mutation negative patients
Figure S2	Survival curve of MEN1 patients, comparing between mutation- positive and mutation- negative index patients
Table S4	Causes of mortality in index cases

Table S1. Age of diagnosis of MEN1 manifestation

MEN1 manifestations	Total		Mutation positive		Mutation negative	
	Median	SD/range	Median	SD/range	Median	SD/range
**pHPT**	34	14 (11 – 78)	33	12 (11 – 68)	51	14 (23 – 78)
**dpNET**	39	14 (13 – 78)	39	14 (13 – 78)	48	17 (23 – 63)
**PIT**	38	15 (15 – 73)	36	14 (15 – 71)	49	13 (23 – 73)
**lung NET**	42	14 (18 – 75)	42	14 (18 – 75)		n.a.
**thymic NET**	43	6 (36 – 60)	43	6 (36 – 60)		n.a.
**gastric NET**	50	14 (34 – 75)	50	14 (34 – 75)		n.a.
**adrenal tumor**	47	12 (21 – 73)	47	12 (21 – 73)	48*	n.a.

pHPT primary hyperparathyroidism
dpNET duodenopancretic NET
PIT pituitary tumor
n.a. not applicable
* only one patient

Table S2: Causes of mortality

Cause of mortality	Total Number of patients (%)*	Mutation positive Number of patients (%)*	Mutation negative Number of patients (%)*
**MEN 1 related causes of death**			
**Duodenopancreatic NET: local or metastatic disease**	17 (31.5)	17 (33.3)	0 (0)
**Thymic NET: local or metastatic disease**	9 (16.7)	9 (17.6)	0 (0)
**Died after operation for MEN1 related causes**	3 (5.6)	3 (5.9)	0 (0)
**Kidney failure due to pHPT**	1 (2.5)	1 (2.6)	0 (0)
**Total**	30 (55.6)	30 (58.8)	0 (0)
**Non MEN 1 related causes of death**			
**Other malignancy****	10 (18.5)	10 (19.6)	0 (0)
**Lung embolism**	2 (3.7)	2 (3.9)	0 (0)
**Cardiovascular**	3 (5.6)	1 (2.0)	2 (67)
**Other non MEN 1 related causes*****	9 (16.7)	8 (15.7)	1 (33)
**Total**	25 (44.4)	21 (41.2)	3 (100)
**Total**	54 (100)	51 (100)	3 (100)

pHPT primary hyperparathyroidism
NET neuroendocrine tumor

* Percentage of the subgroup of deceased patients.
** Metastatic sigmoid colon adenocarcinoma (n=4); sarcoma (n=2) metastatic adenocarcinoma of unknown primary origin (n=1); urine bladder(n=1); metastatic prostate carcinoma (n=1); pancreatic carcinoma (n=1);.
***Myotonic dystrophy (n=1); consequences of demential syndrome (n=1); pneumonia (n=1); subarachnoidal bleeding (n=1); chronic obstructive pulmonary disease (n=1), unspecified (n=4).

Table S3 Clinical characteristics and results of genetic screening

Patient	Manifestations (age)				MEN1 MLPA	CDKN1B	CDKN1B MLPA	AIP	AIP MLPA	Comments
	pHPT	PIT	pdNET	Other						
1	31	x	38		ND	ND	ND	ND	ND	
2	x	28	23		-	-	-	-	-	
3	25	x	25		-	-	-	-	-	
4	23	23	x		-	-	-	-	-	
5	61	61	x		-	-	-	-	-	
6	48	55	x	ADR 48	-	-	-	-	-	
7	64	63	x		-	-	-	-	-	
8	53	35	x		-	mutation	-	-	-	
9	48	35	x		ND	ND	ND	ND	ND	
10	39	31	x		-	-	-	-	-	
11	78	54	x		ND	ND	ND	ND	ND	died
12	60	54	x		-	-	ND	-	ND	
13	42	51	x		-	-	-	-	-	
14	62	54	x		-	-	ND	ND	ND	
15	48	29	x		-	-	ND	-	ND	
16	67	50	x		ND	ND	ND	ND	ND	died
17	x	61	61		-	-	-	-	-	
18	73	73	x		-	ND	ND	ND	ND	dementia
19	40	48	x		ND	ND	ND	ND	ND	
20	50	48	x		-	-	-	-	-	
21	24	x	48		-	-	-	-	-	
22	47	46	x		-	-	-	-	-	
23	62	62	x		-	-	-	ND	ND	
24	56	39	x		-	-	-	-	-	
25	51	51	x		-	-	-	-	-	
26	45	60	x		-	-	-	-	-	
27	45	56	x		ND	ND	ND	ND	ND	
28	63	63	x		ND	ND	ND	ND	ND	died
29	62	x	63		-	-	-	-	-	
30	58	x	57		-	ND	ND	ND	ND	

pHPT	primary hyperparathyroidism
PIT	pituitary adenoma
pdNET	duodenopancreatic neuroendocrine tumor
ADR	adrenal tumor
x	absence of the MEN1-related manifestation
ND	Not Determined
-	Determined, no mutations was detected
mutation	Mutation was detected

Figure S1 Age related penetrance of major manifestations and other MEN1-associated tumors compared between mutation positive index patients and mutation negative patients

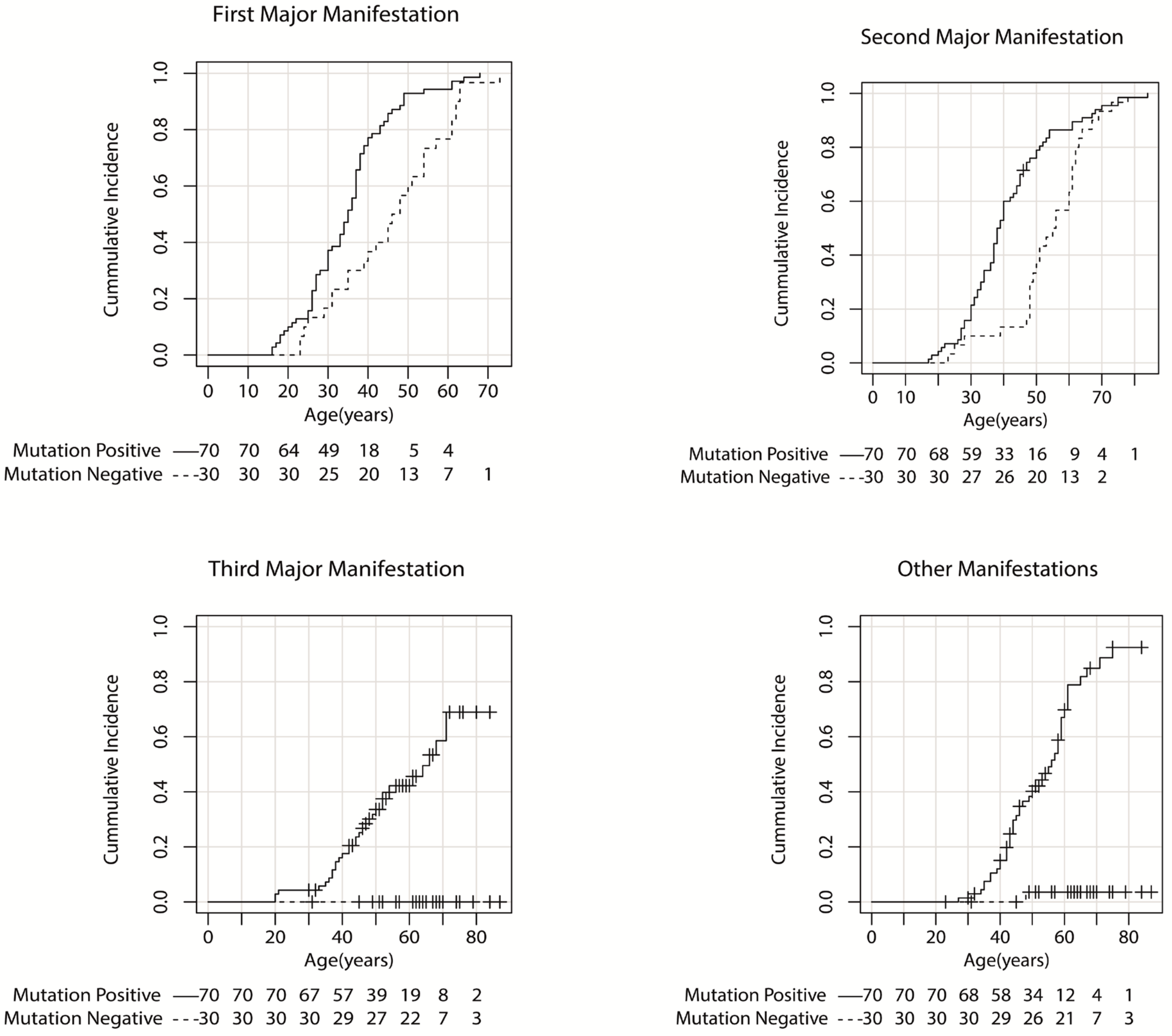

Figure S2 Survival curve of MEN1 patients, comparing between mutation- positive and mutation- negative index patients

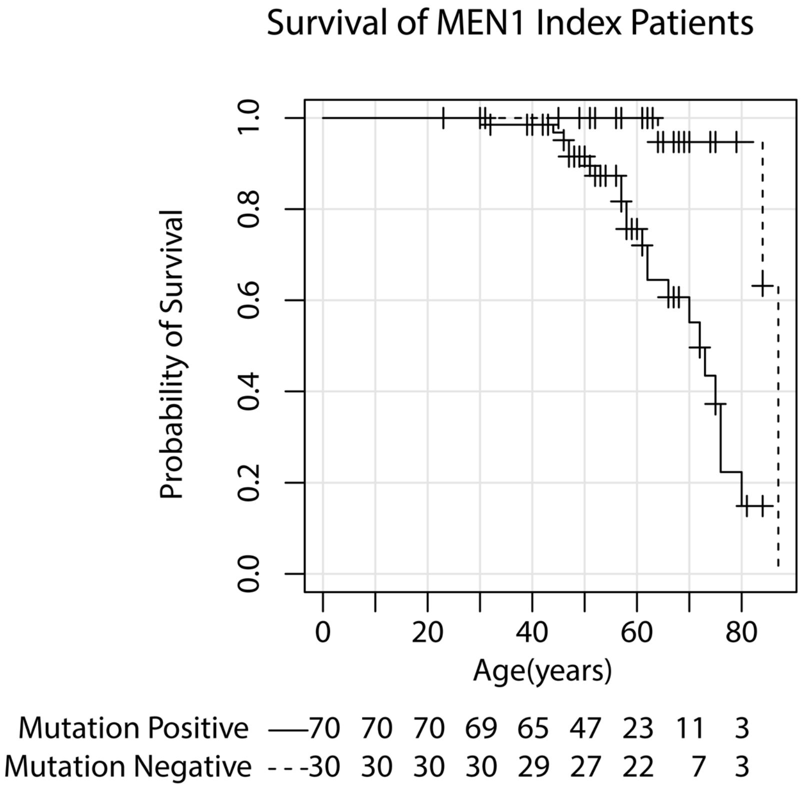

Table S4: Causes of mortality in index cases

Cause of mortality	Total Number of patients (%)*	Mutation positive Number of patients (%)*	Mutation negative Number of patients (%)*
**MEN 1 related causes of death**			
**Duodenopancreatic NET: local or metastatic disease**	11 (44.0)	11 (50.0)	0 (0)
**Thymic NET: local or metastatic disease**	4 (16.0)	4 (18.2)	0 (0)
**Died after operation for MEN1 related causes**	1 (4.0)	1 (45.5)	0 (0)
**Total**	16 (64.0)	16 (72.7)	0 (0)
**Non MEN 1 related causes of death**			
**Other malignancy****	1 (4.0)	1(4.5)	0 (0)
**Lung embolism**	1 (4.0)	1 (4.5)	0 (0)
**Cardiovascular**	2 (8.0)	0 (0.0)	2 (67)
**Other non MEN 1 related causes*****	5 (20.0)	4 (18.2)	1 (33)
**Total**	9 (36.0)	6 (27.3)	3 (100)
**Total**	25 (100)	22 (100)	3 (100)

pHPT primary hyperparathyroidism
NET neuroendocrine tumor

* Percentage of the subgroup of deceased patients.
** Metastatic sigmoid colon adenocarcinoma (n=1);
*** consequences of demential syndrome (n=1); pneumonia (n=1); chronic obstructive pulmonary disease (n=1), unspecified (n=2).
